# Successful minimal-invasive management of misplaced sacroiliac screw perforating the left external iliac vein: an interdisciplinary intervention by angiology and traumatology—a case report

**DOI:** 10.1186/s13256-026-06278-3

**Published:** 2026-06-26

**Authors:** Titus Drossel, Andreas Höch, Markus Doß, Andrej Schmidt, Christian Kleber

**Affiliations:** 1 Department of Orthopedics, Trauma and Plastic Surgery, University Hospital of Leipzig, Liebigstraße 20, 04103 Leipzig, Germany; 2 Department of Visceral, Transplantation, Vascular and Thoracic Surgery, University Hospital of Leipzig, Liebigstraße 20, 04103 Leipzig, Germany; 3 Department of Angiology, University Hospital of Leipzig, Liebigstraße 20, 04103 Leipzig, Germany

**Keywords:** Pelvic trauma, Pelvic ring fracture, Percutaneous sacroiliac screw, Vascular injury, Complication management, Interdisciplinary intervention, Surgical treatment, Case report

## Abstract

**Background:**

Percutaneous sacroiliac screw fixation is an effective option for unstable pelvic ring fractures. It is minimally invasive, causes little soft tissue damage, and reduces complication risk. Nevertheless, complications such as screw loosening or nerve root damage are reported. Although vascular injuries are also described, detailed management strategies are rarely found in the literature.

**Case presentation:**

A 58-year-old white German female suffered polytrauma, including a type c posterior pelvic ring fracture, after a fall from great height. The fracture was treated with screw osteosynthesis of the anterior and posterior ring. After transfer to our hospital for further treatment, X-ray and CT revealed sacroiliac screw malposition with suspected external iliac vein injury. During an interdisciplinary procedure involving angiology and trauma surgery, the screw was removed, and subsequent bleeding from the injured vein was controlled by coiling before a new sacroiliac screw was inserted. The subsequent hospital stay remained complication free.

**Conclusions:**

Sacroiliac screw osteosynthesis is a safe and established procedure for unstable pelvic ring injuries. However, due to complex anatomy such as sacral dysmorphia and difficult fracture patterns, complications including nerve or vascular injury may occur. Preventing these requires careful preoperative planning to identify a suitable corridor. While 2D-fluoroscopy is well established intraoperatively, 3D-navigation further reduces malposition risk in challenging anatomy. In cases of screw malposition, exact analysis and interdisciplinary consultation are essential to determine the safest and most effective treatment and to successfully manage complications.

**Key clinical message:**

The incorrect positioning of a sacroiliac screw can lead to serious complications. Exact analysis and the full spectrum of complication management is necessary for successful treatment of misplaced screws in pelvic surgery.

## Background

Pelvic fractures are classified as a significant clinical concern, particularly when they occur in patients with normal bone density, predominantly as a result of high-impact trauma such as traffic accidents or falls from heights. These injuries can lead to broader clinical complications due to their complex nature [1].

Depending on the fracture morphology, a distinction is made between stable and unstable fractures depending on the integrity of the pelvic ring. A further distinction is made between a vertical sheer and lateral compression fractures [2]. Unstable pelvic fractures necessitate surgical treatment approaches, often employing techniques such as percutaneous iliosacral screw fixation. Due to the percutaneous nature of the procedure, this surgery is a good option over open procedures, as its minimally invasive nature reduces soft tissue damage and minimizes complications and postoperative pain [3]. Nevertheless, inadequate placement of the IS-screw (ISs) can lead to significant complications. These include, for example, screw loosening or neurovascular damage. There are individual reports in the literature of damage to nerve roots caused by S2 screw connections [4]. While literature highlights potential complications, there is limited extensive reporting on vascular damage stemming from misplaced screws, indicating a knowledge gap in this aspect.

In this case report, we report the challenge of detection and unique minimal-invasive management of a patient with misplaced ISs whose insertion resulted in injury to the left external iliac vein.

### Case presentation

We report on a case of a 58-year-old German female patient who fell from a great height while hiking on Lions Head in South Africa and suffered polytrauma. This includes a hemothorax with fracture of the first rib, a sternal fracture, a fracture of the 2nd lumbar vertebra, as well as an ankle fracture on the right, an olecranon fracture on the left, and the ipsilateral anterior and posterior pelvic ring type c fracture on the right.

After intubation and transportation by rescue helicopter to a hospital, the fractures were treated. This included screw osteosynthesis of the anterior (retrograde) and posterior pelvic ring fracture (hexacortical ISs). Once the patient's condition had stabilized, she was transferred to our clinic for further treatment.

**Fig. 1:**
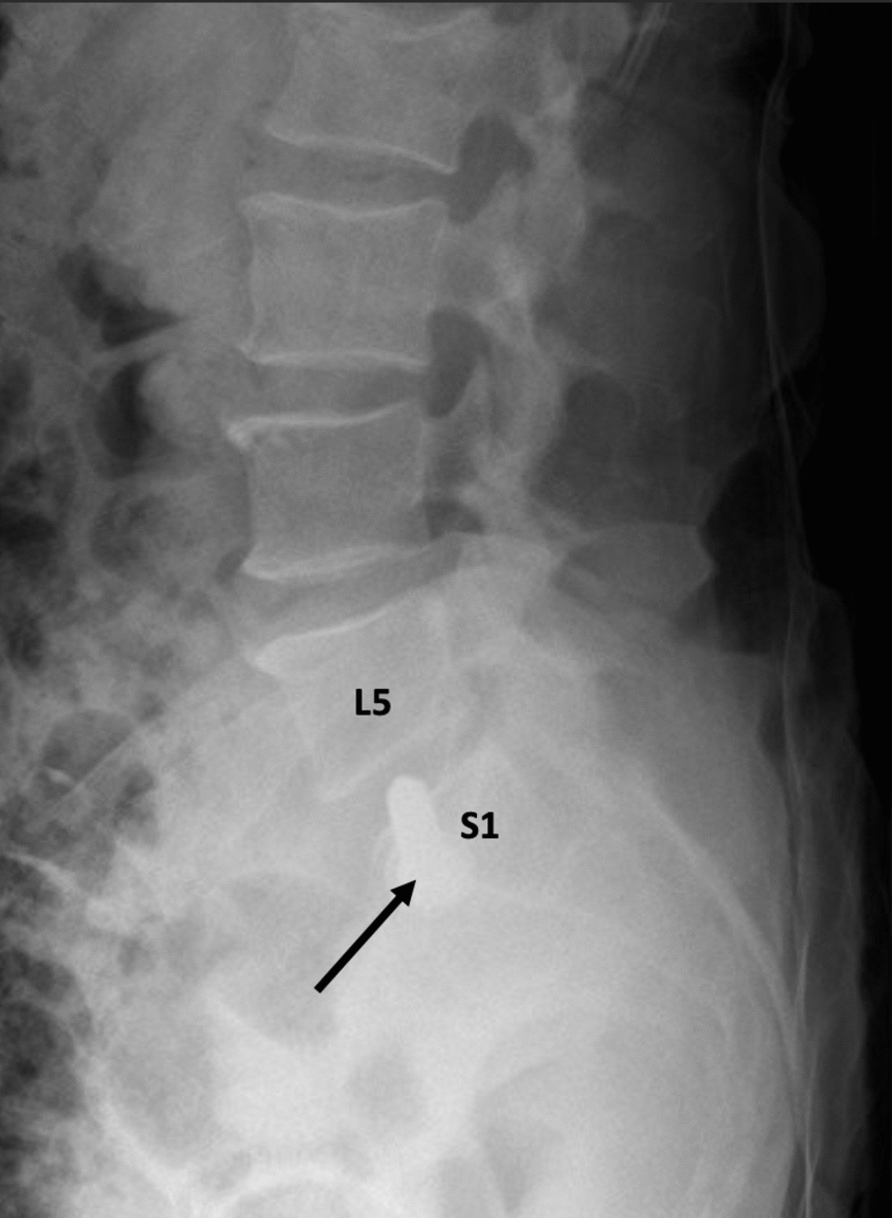
X-ray of the lumbar spine and the sacrum of the patient. L5: fifth lumbar vertebra, S1: first sacral vertebra. The arrow points to the misplaced ISs

After arrival of the patient and examination of the X-ray images provided, a screw malposition of the posterior pelvic screw was strongly suspected due to crossing the ala-slope of the screw in the lateral view (Fig. 1).

**Fig. 2:**
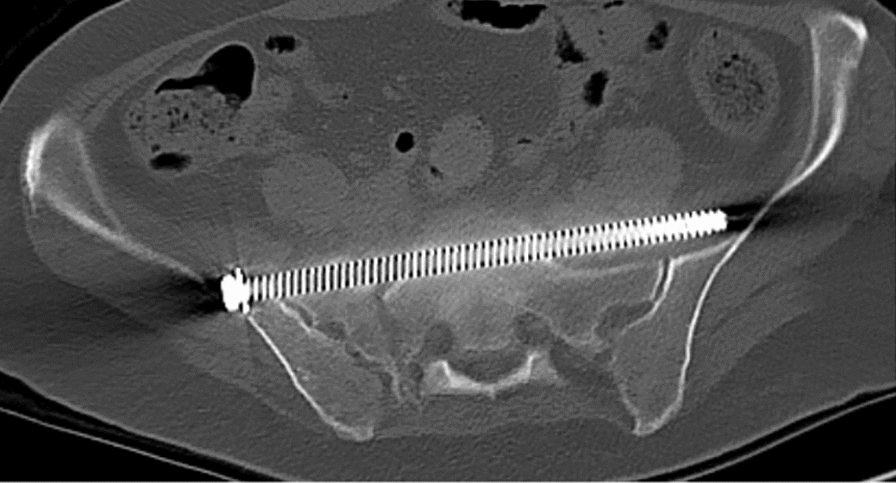
CT scan of the patients’ pelvic bones showing the misplaced screw

For this reason, a native CT scan was performed. The CT scan showed that the ISs was far anterior and mostly outside the bone (Fig. 2).

**Fig. 3:**
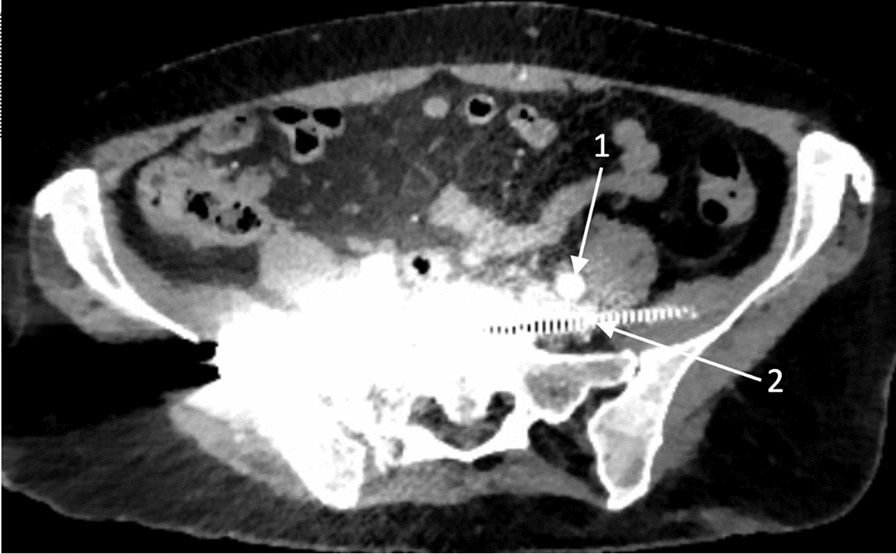
Visualization in the soft tissue window, showing the proximity to the external iliac vein, 1: left common iliac artery, 2: SI screw compressing the left external iliac vein

**Fig. 4:**
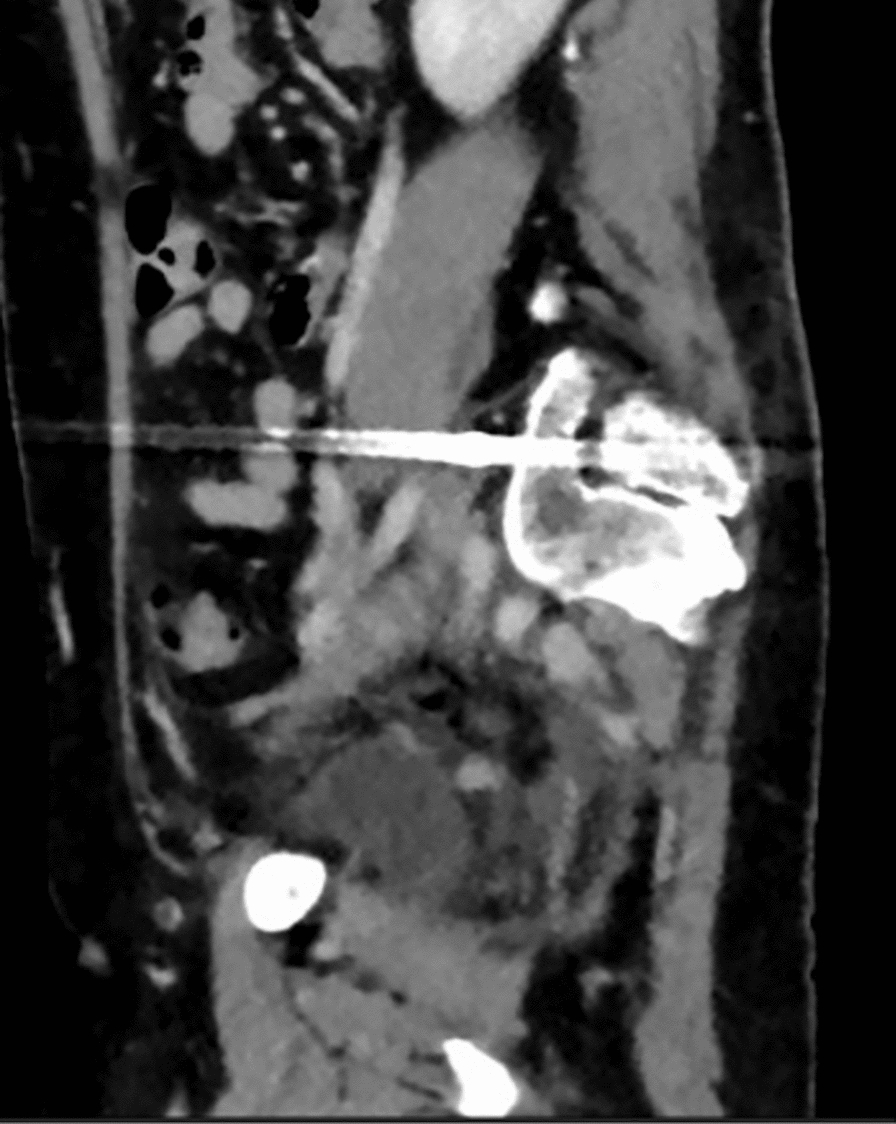
Sagittal view of the misplaced sacroiliac screw

Another cause for concern was the proximity of the ISs to the iliac vessels on the left side, so that there was a strong suspicion that the screw could have touched the vein and possibly even injured it. Therefore, we repeated the CT scan with contrast to reveal a vessel injury as shown in Figs. 3 and 4.

Again, we could proof the direct contact of the vessels to the screw but no clear evidence for injury of the vessels or even perforation. Furthermore, the suspicion of injury to the iliac vein was given by the fact that the patient presented with a 4-level thrombosis in her left leg confirmed by duplex compression ultrasound. This made it clear that the screw must have either injured or compressed the vein.

In the light of the complexity of this case, which resulted from the combination of a trauma surgery, vascular surgery, and angiology problem, an interdisciplinary consultation was held between the traumatology, vascular surgery, and angiology departments.

### Decision making

After intensive consultations, the following treatment options were offered:

1. Simple removal of the screw while the patient is on standby for vascular surgery carries the risk of uncontrolled and potentially life-threatening bleeding in case of iliac vascular injury.
2. Open exposure of the potentially injured iliac vessels and removal of the screw under visualization. In view of the significant invasive nature of the procedure, high surgical approach morbidity would be expected here. And in case of no vascular injury unnecessary laparotomy would have been performed.
3. Implementation of a blocking catheter by interventional angiology, followed by removal of the screw under angiographic monitoring to identify potential bleeding.
4. The last option was to leave the screw in its incorrect position with the enormous risk of potentially spontaneously occurring uncontrollable bleeding, persisting back pain, and pseudarthrosis.

After a comprehensive evaluation of the risks and benefits, the decision was made within the multidisciplinary team to choose the third option with removal of the screw under angiographic visualization. The decisive advantage of this method is the reduced invasiveness of the procedure while at the same time ensuring the identification of bleeding.

### Minimal-invasive management

#### 1. Phlebography and IVC implantation

**Fig. 5:**
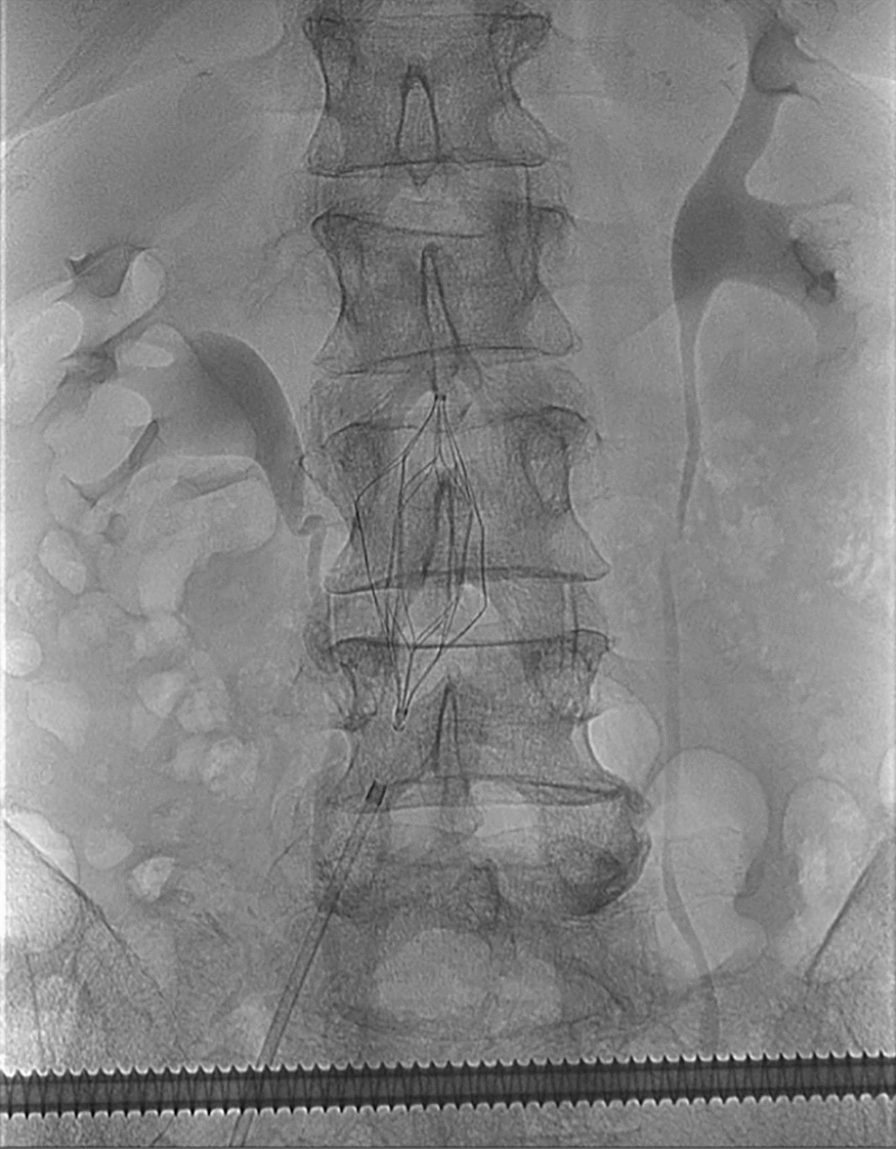
Insertion of the IVC filter before surgery

Prior to the surgical procedure, phlebography was performed to determine the position of the constriction. Due to the deep vein thrombosis, a retrievable inferior vena cava (IVC) filter (Optease^™^; Cordis) was inserted to minimize the risk of a potentially life-threatening pulmonary embolism during the interventional treatment as presented in Fig. 5. In order to detect injury of the vein an IVUS (intravascular ultrasound) was performed. Unfortunately, even here the differentiation between vein wall injury and compression was not quite clear.

#### 2. Combined surgical and angiological procedure

**Fig. 6:**
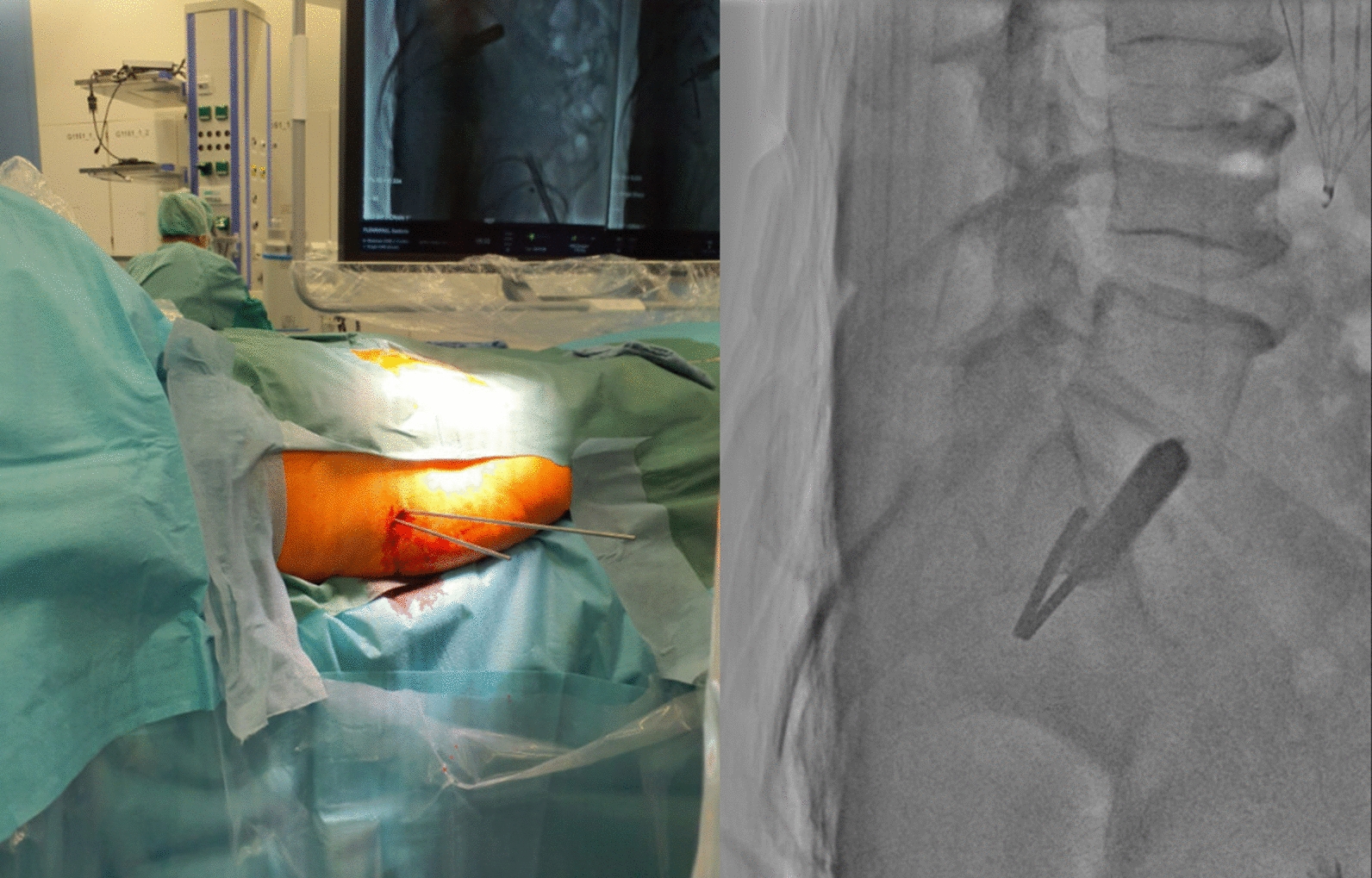
Insertion of a guide wire for the new SI screw

The minimal-invasive management was performed in a hybrid-operation room with Siemens Artis Pheno VE20 system. After careful bedding with access to the abdomen, bilateral sacrum, and groins a guide wire was inserted into the misplaced SI screw at the start of the operation to enable subsequent removal via cannulated screwdriver. A second guide wire was then positioned under X-ray control in the first sacral body for the new SI screw to be inserted. An intraoperative photo and an X-ray check of the wire are shown in Fig. 6. This revealed that the guide wire for the new SI screw could not be drilled over as it was too close to the washer of the previously incorrectly positioned screw. Due to the present situation, the angiology colleagues now cannulated both vena femoralis and inserted two guide wires to introduce balloons for blocking the iliac veins.

The first step was a crossover maneuver from the right femoral vein into the left common iliac vein and then into the left internal iliac vein. The second blocking balloon catheter was inserted retrograde on the left into the junction between the common iliac vein and the external iliac vein through the thrombotic vessel.

**Fig. 7:**
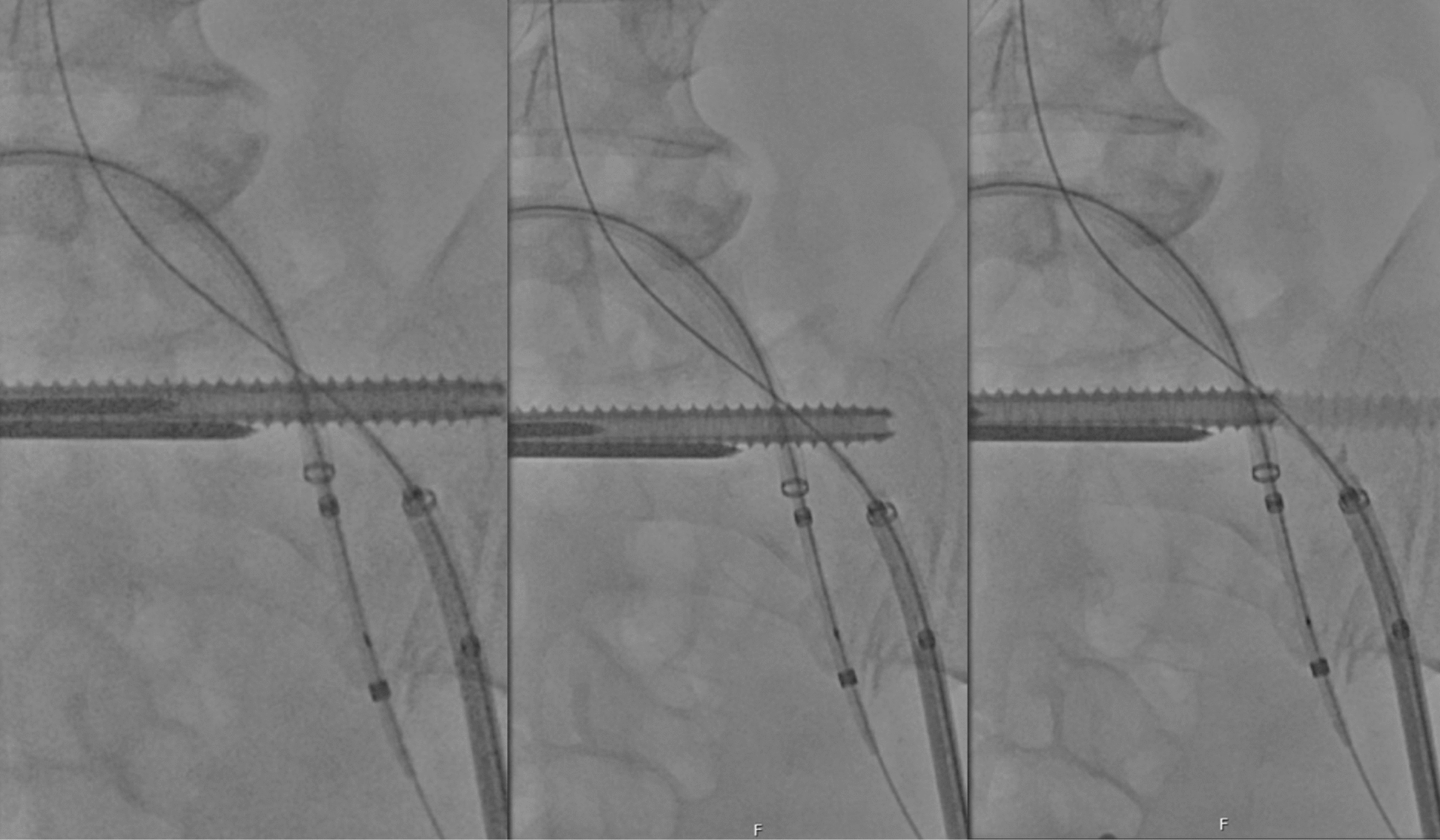
Angiologic representation of the screw channel and the removal of the SI screw

**Fig. 8:**
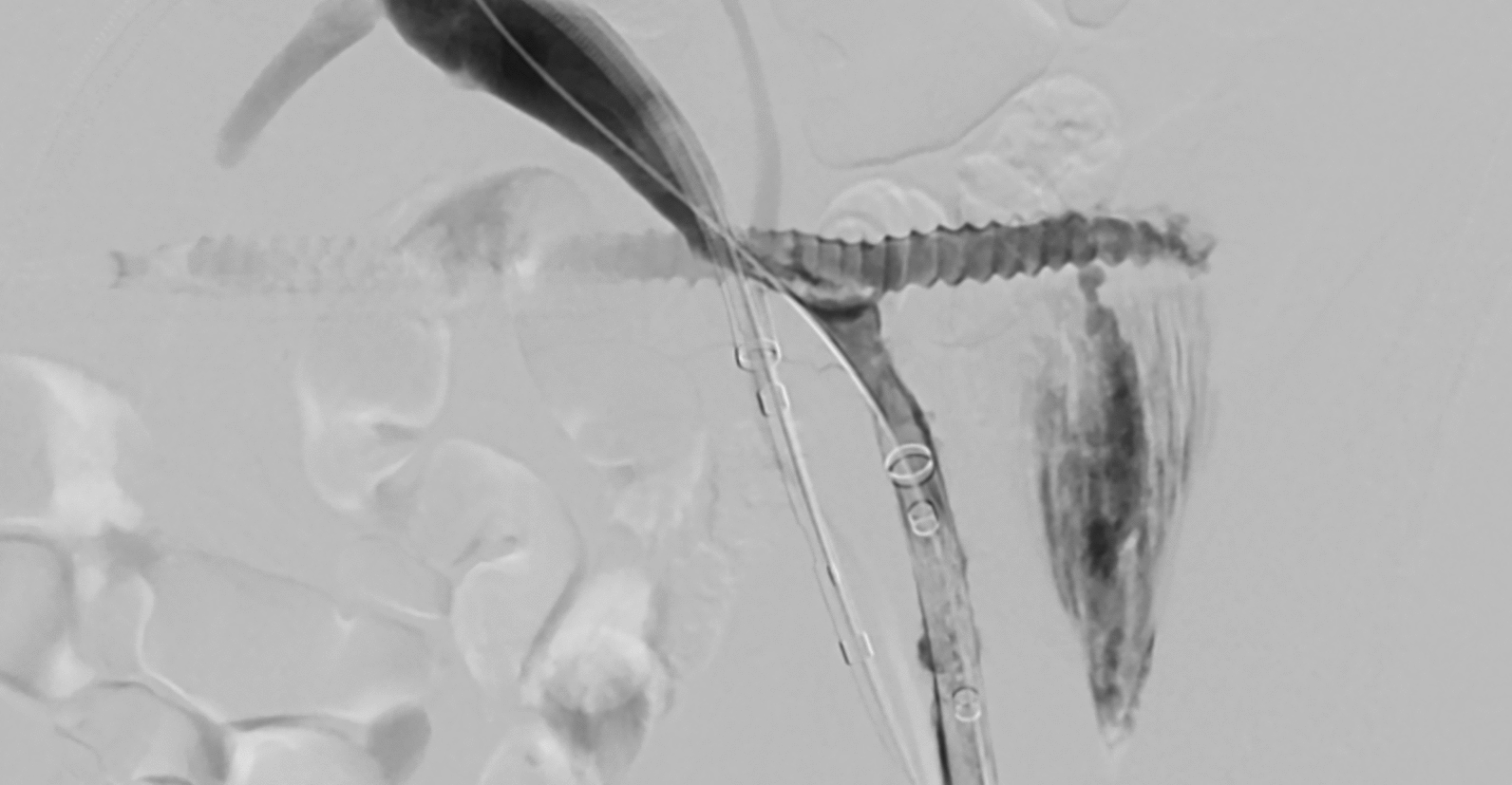
After screw removal, the screw channel filled with contrast medium and bleeding into the muscle occurred

**Fig. 9:**
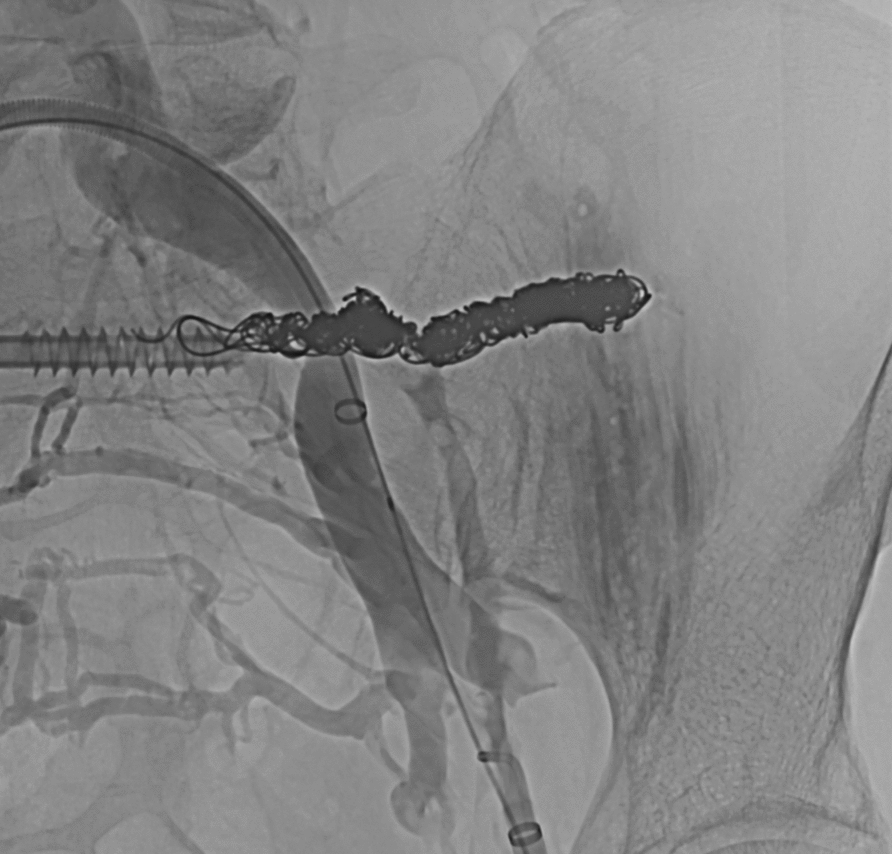
Bleeding into the screw channel and muscle ceased completely after coil embolization of the channel

Under X-ray control a careful removal of the misplaced ISs was performed. An image series of the removal is shown in Fig. 7. Once the tip of the screw crosses the iliac vein contrast spill was observed in the former screw canal. At the same time, there was massive bleeding from the sacral bone. This was quickly stopped with Ethicon TABOTAMP® and bone wax. A significant leakage of the contrast medium into the former screw channel and the iliopsoas muscle from the external iliac vein was detected and is presented in Fig. 8. This observation supports the hypothesis that the screw had perforated the vein and that the screw itself tamponed the vessel. The screw channel was closed to the left and right using a total of 15 coils (Cook™ Nester and Penumbra^™^ Coils 6/7 mm) as shown in Fig. 9. As a result, no more contrast medium accumulation could be detected in this area. The channels were consequently closed.

Once the bleeding situation was under control, the new sacroiliac screw could be inserted under X-ray control.

The minimal-invasive procedure was finished in 132 min with 300 ml of blood loss.

Due to the iliac vessel injury, we decided to insert an indicator drain at the insertion of the ISs. Due to the bleeding from the psoas and sacrum, postoperative monitoring was carried out in the intensive care unit.

**Fig. 10:**
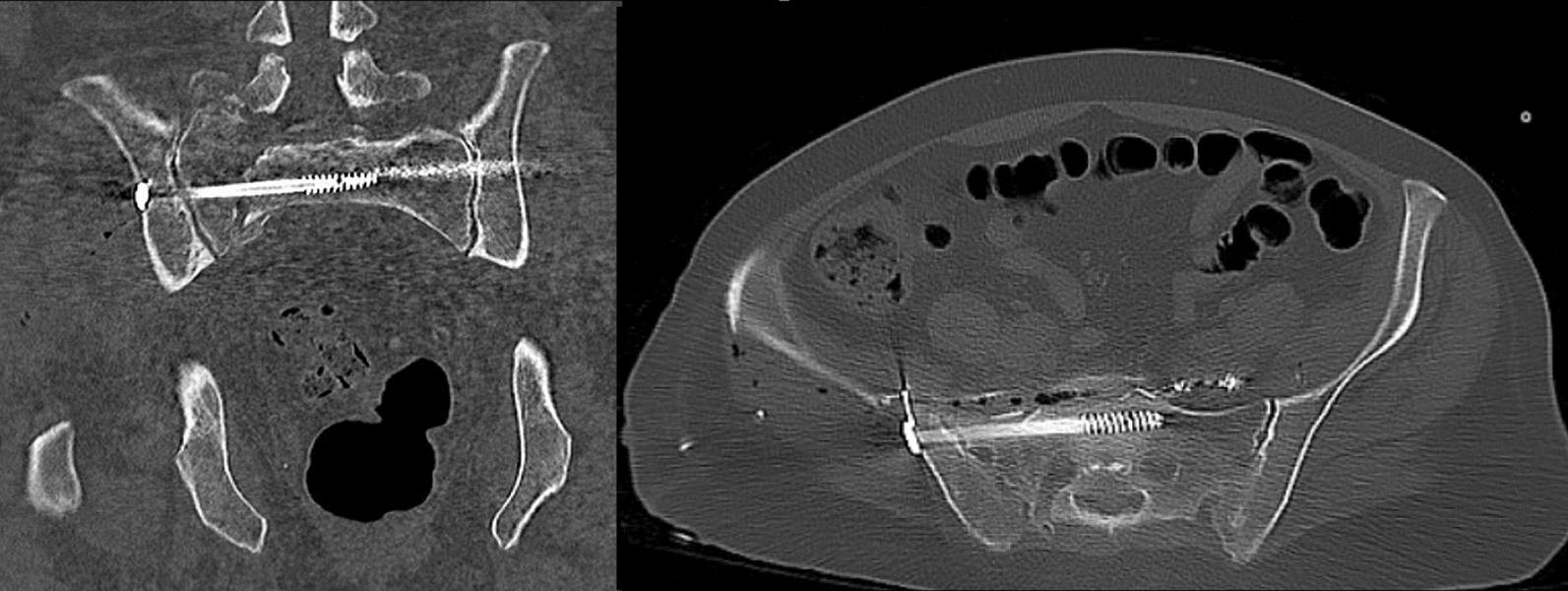
Postoperative CT scan confirming the correct position of the ISs

**Fig. 11:**
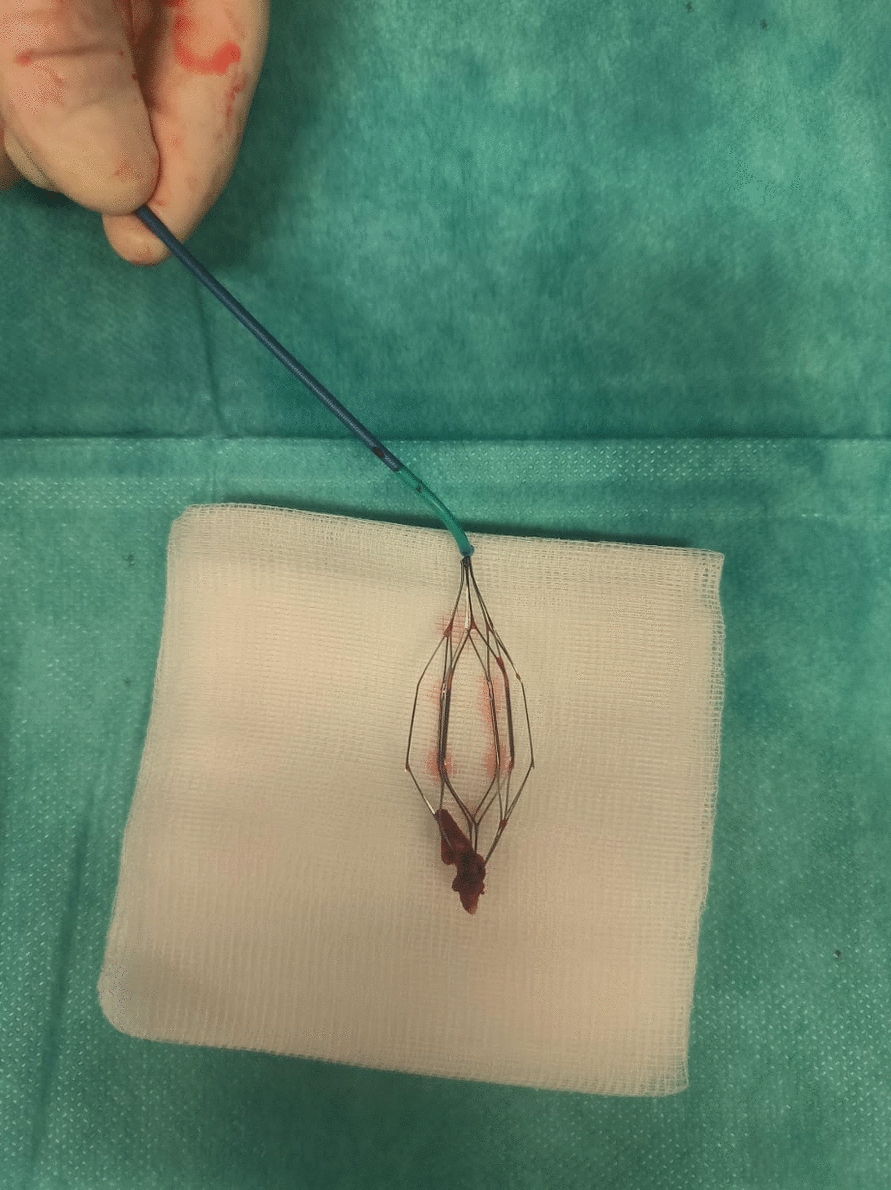
The removed inferior vena cava (IVC) filter with thrombus attached to the lower end

A postoperative CT scan on the next day showed the correct position of the new inserted ISs and no active bleeding (Fig. 10). The inserted IVC was removed on the 5th postoperative day. An organized thrombus was found in the filter as shown in Fig. 11.

**Fig. 12:**
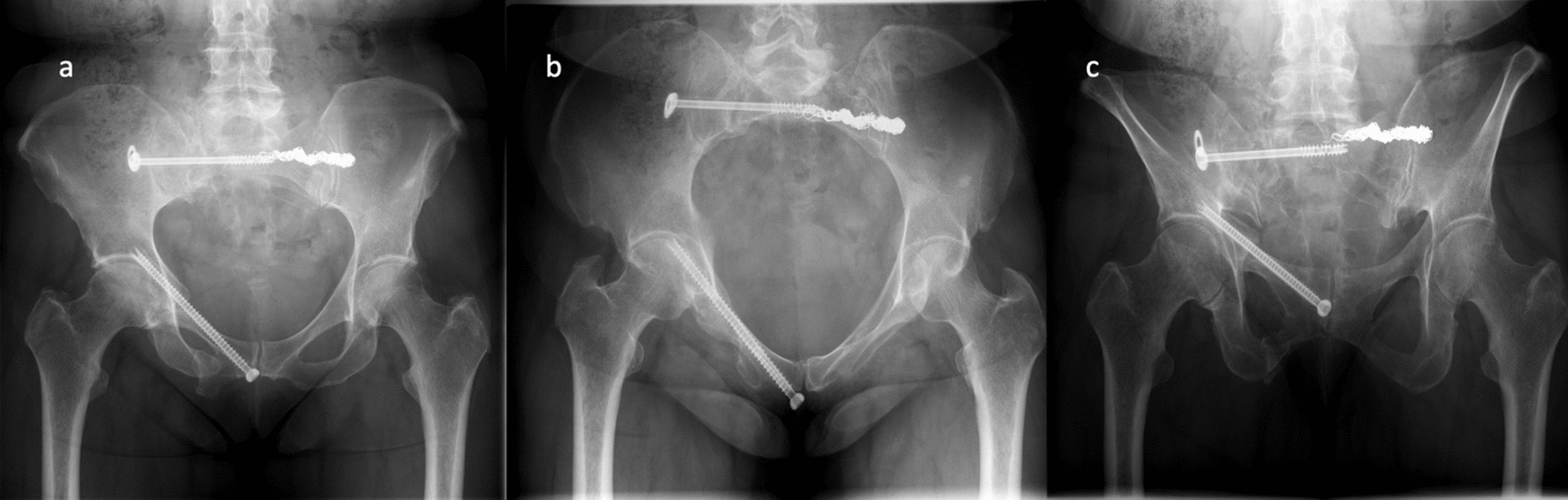
Nine-month follow-up X-rays, showing a pelvic X-ray **a**, as well as inlet **b** and outlet **c** view

This underlines the immense importance of the IVC in operations with concomitant venous thrombosis, as a life-threatening pulmonary embolism was prevented. The patient was able to leave the intensive care unit after 1 day. A revision of the plate osteosynthesis on the ankle joint was carried out during the further course of the inpatient stay. The patient was then discharged in good general condition just 10 days after the pelvic operation. In the post-hospital follow-up, the screw was found to be in the correct position with no signs of possible dislocation or loosening (Fig. 12).

During her inpatient stay, the patient initially reported feelings of stress relating to the revision surgery and the potential for complications. The patient’s positive outlook was influenced by a comprehensive explanation of the procedure and a detailed description of the intensive interdisciplinary discussion of the case. Postoperatively, there was a rapid improvement in mobility. In the course of regular follow-up examinations, the patient expressed satisfaction with the overall course of treatment and reported an improvement in mobility and pain.

## Discussion

The treatment of unstable posterior pelvic ring fractures using iliosacral screw fixation is an established and safe procedure. While open reduction and internal fixation were favored or utilized with equal regularity in the early 2000 s and prior, there has been a discernible shift toward percutaneous internal fixation [5]. The primary rationales for this phenomenon pertain to the reduction in blood loss and the diminished probability of wound infections [6]. Nevertheless, complications can also occur in this frequently performed procedure due to complex anatomy. Misplacement of an iliosacral screw in neurovascular structures, such as the one described in this case in the external iliac vein, is a rare but serious and potentially life-threatening complication. Apart from analyzing the complication and treating it accordingly, it is crucial to develop possible strategies to prevent such misplacements. This includes preoperative planning as well as intraoperative and postoperative imaging.

One of the most important measures to avoid misplacement is precise and detailed preoperative planning using imaging techniques. The correct positioning of the iliosacral screw depends heavily on the exact determination of the anatomical structure and fracture localization.

Meticulous preoperative planning is crucial, particularly in patients with sacral dysmorphism, which affects up to 58% of adults [7]. Dysmorphic sacra exhibit variations in the alar slope, foramina positioning, and vertebral morphology, all of which necessitate careful adjustment of screw orientation. The variability of the S1 vertebra also poses a challenge for the placement of the screw. As early as 1997, Ebraheim et al used morphological analysis of the S1 vertebra to provide important findings regarding a safe corridor for placement of the screw and the correct choice of entry point. [8].

Before surgery, a detailed CT scan of the pelvis is essential to enable three-dimensional visualization of the bone and surrounding soft tissue structures. This enables optimal surgical planning regarding possible treatment strategies, access routes, and possible pitfalls such as proximity to neurovascular structures.

In CT analyses, Hoppler et al found that in bilateral fragility fractures, the S1 segment only showed a suitable corridor with a width of > 8 mm in 80% of the cases examined. The S2 segment only offered this in 47% of cases. This is particularly important for assessing whether an SI screw can be inserted at all [9]

Despite preoperative planning and surgical expertise, unexpected anatomical variations or intraoperative challenges may occur. For this reason, continuous monitoring of screw placement during the procedure is essential.

Historically, screw placement has been performed under 2D fluoroscopic guidance, relying on standard inlet, outlet, and lateral views to navigate the sacral anatomy. This technique was utilized in the 1980 s and 1990 s for percutaneous fixation of pelvic injuries. In 1989, Matta originally described the internal fixation of the pelvic ring in the prone position as [10]. Subsequent studies by Routt et al. demonstrated that percutaneous fixation in the supine position was also feasible, thus offering a more straightforward positioning method that enabled simultaneous operations and concomitantly reduced operating and anesthetic times [11]. Moreover, the infection rate associated with the percutaneous technique was found to be exceptionally minimal [12].

While effective in many cases, 2D fluoroscopy is inherently limited by its inability to provide axial visualization, which can result in a substantial rate of screw misplacement. Reported malposition rates with 2D fluoroscopy range from 2% to as high as 15%, depending on patient anatomy, surgeon experience, and image quality [13]. These misplacements can lead to serious complications such as the previous mentioned injury to the lumbosacral nerve roots, vascular injury––particularly to the internal iliac vessels or superior gluteal artery––and suboptimal fracture stabilization.

The advent of 3D navigation systems, including intraoperative CT and computer-assisted guidance, has dramatically improved the precision of SI screw placement. With real-time, multiplanar imaging and enhanced visualization of individual sacral morphology, 3D systems allow for trajectory planning that avoids neural foramina and vascular structures. Studies have shown that using 3D navigation can reduce malposition rates to as low as 0–5% [14, 15]. In comparative studies, patients treated with 3D navigation had significantly fewer complications, and screw repositioning was rarely required [8].

Moreover, clinical outcomes appear to be superior with 3D navigation, as accurate screw placement correlates with better biomechanical stability and reduced rates of revision surgery. The ability to safely insert longer screws with optimal orientation enhances construct stability, which is particularly important in vertically unstable injuries. Despite these benefits, factors such as equipment cost, availability, the need for training, and time delay due to the setup initialization of the navigation system must be considered when implementing 3D navigation technology.

In conclusion, while 2D fluoroscopy remains widely used and effective in skilled hands, 3D navigation offers superior accuracy in iliosacral screw placement and significantly reduces the risk of neurovascular complications. Its application is particularly advantageous in cases involving sacral dysmorphism or complex fracture patterns, where precise screw trajectory is essential for both safety and optimal fixation [16].

Despite detailed preoperative and intraoperative planning, complications can still arise during surgery.

The most clinically relevant complications of SI screw placement are neurovascular in nature. As little as 4° of deviation from the ideal screw trajectory may lead to perforation of the sacral foramina or the ventral sacral cortex [17]. Reported rates of screw malposition reach up to 24%, and neurological complications may occur in up to 18% of cases [17]. These include injuries to the lumbosacral trunk, internal iliac vessels, and the superior gluteal artery. Notably, even correctly placed screws can endanger the superior gluteal artery due to its proximity to the screw trajectory [17, 18].

Other complications include malreduction and malunion, with posterior displacement of the pelvic ring > 1 cm being significantly associated with chronic pain and poor functional outcomes [3]. Implant failure and nonunion are less frequent but clinically relevant, especially in osteoporotic bone or in cases with suboptimal reduction. Deep infections are rare but may require surgical debridement and implant removal, whereas superficial infections are usually managed conservatively [17]. Surgical experience is also a significant factor influencing the complication rate. Several studies have demonstrated that, even when utilizing 3D fluoroscopic imaging, there is an increased incidence of misplacement when the surgical technique and anatomical knowledge are at a lower level (Alviset al 2014; Konrad et al 2010) [19, 20].

If a screw is nevertheless misplaced, a precise analysis of the situation at hand is essential. This is particularly important regarding possible risks, such as severe bleeding due to vascular injury. In such cases, a rapid but carefully considered approach is required. Moreover, it is not possible to formulate a universal statement regarding the management of misplaced screws; rather, this decision must be made on an individual patient basis. A plethora of case reports have documented instances of nerve damage following open or percutaneous screw removal, with the decision to perform either procedure contingent on the integrity of the screw [21]. Interdisciplinary collaboration is of significant relevance here to open up a wide range of options and determine the most effective and least risky option. An example of this is the decision between vascular surgery and interventional therapy to stop bleeding, as in this case.

## Conclusion

The incorrect positioning of a sacroiliac screw can sometimes lead to serious complications. Therefore, the use of intraoperative 2D/3D imaging and navigation should pose a standard in pelvic surgery, especially in complex sacral anatomy or dysmorphism. Exact analysis and the full spectrum of complication management is necessary for successful treatment of misplaced screws in pelvic surgery. We present a successful interdisciplinary and minimal-invasive approach for revision of misplaced ISs and iliac vessel injury.

## Data Availability

The datasets used during the current study are available from the corresponding author on reasonable request.

## Abbreviations

ISs: Iliosacral screw
IVC: Inferior vena cava
CT: Computer tomography
S1: First sacral vertebra
S2: Second sacral vertebra
